# Investigating local and systemic intestinal signalling in health and disease with *Drosophila*

**DOI:** 10.1242/dmm.049332

**Published:** 2022-03-28

**Authors:** Andre Medina, Karen Bellec, Sofia Polcowñuk, Julia B. Cordero

**Affiliations:** 1 Wolfson Wohl Cancer Research Centre, Institute of Cancer Sciences, University of Glasgow, Garscube Estate, Switchback Road, Glasgow G61 1QH, UK; 2 CRUK Beatson Institute, Institute of Cancer Sciences, University of Glasgow, Garscube Estate, Switchback Road, Glasgow G61 1BD, UK

**Keywords:** *Drosophila*, Intestine, Disease, Health, Inter-organ communication

## Abstract

Whole-body health relies on complex inter-organ signalling networks that enable organisms to adapt to environmental perturbations and to changes in tissue homeostasis. The intestine plays a major role as a signalling centre by producing local and systemic signals that are relayed to the body and that maintain intestinal and organismal homeostasis. Consequently, disruption of intestinal homeostasis and signalling are associated with systemic diseases and multi-organ dysfunction. In recent years, the fruit fly *Drosophila melanogaster* has emerged as a prime model organism to study tissue-intrinsic and systemic signalling networks of the adult intestine due to its genetic tractability and functional conservation with mammals. In this Review, we highlight *Drosophila* research that has contributed to our understanding of how the adult intestine interacts with its microenvironment and with distant organs. We discuss the implications of these findings for understanding intestinal and whole-body pathophysiology, and how future *Drosophila* studies might advance our knowledge of the complex interplay between the intestine and the rest of the body in health and disease.

## Abstract (precis)

**Summary:** We outline work in the fruit fly *Drosophila melanogaster* that has contributed knowledge on local and whole-body signalling coordinated by the adult intestine, and discuss its implications in intestinal pathophysiology and associated systemic dysfunction.

## Introduction

The adult intestine of many metazoan animals is a highly regenerative epithelium that acts as a barrier and as a central coordinator of organismal physiology. These vital roles of the intestine are achieved via local interactions with its microenvironment and long-range communication with distant organs. To improve our knowledge of the mechanisms that mediate local and systemic intestinal signals, researchers need a genetically amenable *in vivo* model system in which to study the intestine in the context of its natural microenvironment and as part of a multi-organ complex. Such a model would allow a better understanding of intestinal pathophysiology and its systemic consequences.

The fruit fly *Drosophila melanogaster* has been successfully used as a model organism to study general principles of physiology and disease (Biteau et al., 2011; Naszai et al., 2015; Bilder et al., 2021). Its ‘simpler’ and highly conserved organ system, combined with the availability of superlative genetic tools and biochemical, metabolic and behavioural assays, have positioned the fruit fly as a unique *in vivo* platform for discovery research and for re-examining long-standing, poorly understood biological phenomena.

**Fig. 1.**
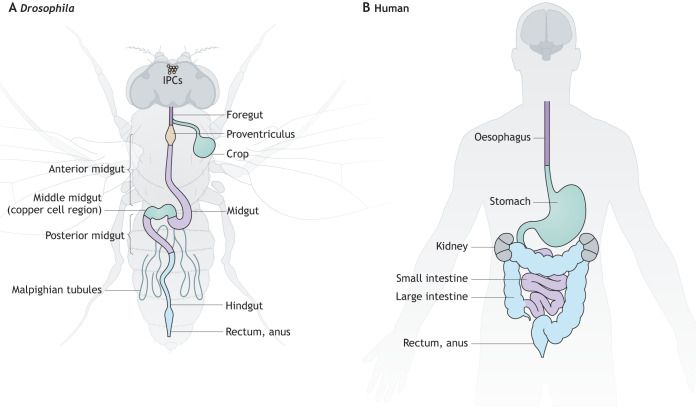
**Comparison of gastrointestinal tract anatomy between adult *Drosophila melanogaster* and humans.** (A,B) The adult *Drosophila* (A) and human (B) gastrointestinal tracts share structural and functional homology. Organs and/or tissues that share the same physiological functions are represented in the same colour. In A, top is anterior and shows the location of the insulin-producing cells (IPCs). The copper cell region (Box 1) and crop are shown in the same colour to reflect that both structures share functional similarities with the human stomach.

Studies in the adult *Drosophila* gastrointestinal tract, which shares structural and functional homology with the mammalian gastrointestinal system (Fig. 1A,B), have shed light on multiple cellular and molecular processes that contribute to intestinal homeostasis, regeneration and tumourigenesis (Jiang et al., 2016; Colombani and Andersen, 2020; Bilder et al., 2021), and that mediate the regulation of host immunity (Ferguson and Foley, 2021), metabolism (Kim et al., 2021b) and behaviour (Cai et al., 2021; Hadjieconomou et al., 2020) by the intestine.

The adult fly gut consists of an epithelial monolayer that forms a cylindrical structure divided into three main regions: the foregut, the midgut and the hindgut (Fig. 1A) (Miguel-Aliaga et al., 2018). The foregut encompasses the pharynx, the oesophagus and the crop (see Glossary, Box 1), an organ involved in food storage. The midgut, akin to the mammalian small intestine, extends from the cardia (Box 1) to the junction with the hindgut, where the Malpighian tubules (Box 1) (Dow and Davies, 2001) connect to the gut. The hindgut, similarly to the mammalian large intestine, fulfils the excretory functions of the fly gastrointestinal system.

To counteract the loss of epithelial cells, the *Drosophila* midgut epithelium relies on the self-renewing capacity of intestinal stem cells (ISCs) to maintain basal tissue homeostasis and to repair the intestinal epithelium upon damage. Following division, each ISC gives rise to a new ISC and to a progenitor cell, either an enteroblast (EB; Box 1) (Ohlstein and Spradling, 2006; Micchelli and Perrimon, 2006) or a pre-enteroendocrine cell (pre-EE cell; Box 1) (Zeng and Hou, 2015; Li et al., 2017; Guo and Ohlstein, 2015; Chen et al., 2018). EBs differentiate into nutrient-absorbing enterocytes (ECs; Box 1) in a Notch signalling (Box 1)-dependent manner (Micchelli and Perrimon, 2006; Ohlstein and Spradling, 2006), while pre-EE cells differentiate into hormone-secreting enteroendocrine (EE) cells (Box 1) (Zeng and Hou, 2015; Beehler-Evans and Micchelli, 2015). Although the *Drosophila* intestinal epithelium possesses a simpler stem cell lineage than that of mammals, the overall cellular functions and molecular principles that dictate ISC proliferation and differentiation are highly conserved between flies and mammals. These similarities include, for example, the origin and function of Wnt and epidermal growth factor (EGF)-like ISC niche (Box 1) components (Buchon et al., 2010; Biteau and Jasper, 2011; Jardé et al., 2020; Perochon et al., 2018), and the role of Wnt, Src and Hippo signalling in adult intestinal regeneration and tumourigenesis (Cordero et al., 2014; Gregorieff et al., 2015; Guillermin et al., 2021; Kohlmaier et al., 2015; Ren et al., 2010; Shaw et al., 2010; Staley and Irvine, 2010; Taniguchi et al., 2015; Yui et al., 2018; Perochon et al., 2018).

Here, we review *Drosophila* research on local and whole-body signalling that is coordinated by the adult intestine and how this research informs our understanding of intestinal pathophysiology and its systemic implications. We focus primarily on work on the adult midgut, which is the best functionally characterised compartment of the *Drosophila* gut.

**Box 1. Glossary**

• **Angiocrine factors:** endothelial cell-derived secreted molecules that stimulate organ growth and remodelling in homeostatic conditions or upon damage/pathology.

• **Bursicon-α (Burs):** insect-specific neuropeptide hormone expressed in enteroendocrine cells and neuronal cells.

• **Cardia:** also known as proventriculus; a structure at the junction between the foregut and midgut where the midgut and the crop merge. It functions as a valve to allow the passage of food into the anterior midgut and crop.

• **Copper cell region:** acidic region in the middle midgut. Together with the crop, this structure is often referred to as the fly ‘stomach’.

• **Corpora cardiaca:** neuroendocrine tissue functionally analogous to human pancreatic α-cells. In *Drosophila*, it is located at the side of the aorta and produces Adipokinetic hormone (Akh), a glucagon-like molecule.

• **Crop:** an enlarged structure in the foregut suggested to have a role in food storage, digestion and microbial control.

• **Dysbiosis:** disruption or alteration of the gut microbiota homeostasis.

• **Endothelial tip cells:** leading cells in the mammalian vascular system that are located at the tip of vascular sprouts and play a key role in angiogenesis. Endothelial tip cells are highly plastic and regulate vascular remodelling.

• **Enteroblasts (EBs):** intestinal progenitor cells derived from intestinal stem cell division that are able to differentiate into absorptive enterocytes.

• **Enterocytes (ECs):** intestinal absorptive cells with a simple columnar epithelial shape. ECs secrete digestive enzymes and are involved in the absorption and transport of nutrients.

• **Enteroendocrine (EE) cells:** intestinal secretory cells that arise from the differentiation of pre-enteroendocrine cells. EEs are best known for producing peptide hormones, which are secreted into the circulation and regulate the function of distant organs within the body.

• **Immune deficiency (IMD) pathway:** an innate immune pathway known to regulate the activity of the *Drosophila* NF-κB-like protein Relish and the production of anti-microbial peptides.

• **Intestinal stem cell (ISC) niche:** the specific intestinal microenvironment that controls ISC behaviour.

• **Malpighian tubules:** pair of tubular structures in arthropods, at the junction between the midgut and the hindgut, which fulfil excretory functions comparable to those of the mammalian kidneys.

• **Myosuppressin (Ms):** a muscle function-inhibiting peptide expressed mainly in neuronal cells.

• **Notch signalling:** a conserved intercellular communication pathway involved in a wide range of cellular processes, such as cell fate specification, cell differentiation and cell proliferation.

• **Pre-enteroendocrine (pre-EE) cells:** ISC progeny committed to becoming an EE cell after differentiation.

• **Target of rapamycin (Tor):** an evolutionarily conserved kinase that promotes cell and tissue growth by coupling growth factors to nutrient availability.

• **Visceral muscle:** muscle that surrounds the epithelium of the gastrointestinal tract and is involved in the peristalsis process, a wave-like muscular contraction important for digestion, pathogen clearance and the transport of ingested food along the intestinal tract.

## Intestinal–microenvironment interactions in *Drosophila*

The mammalian intestinal epithelium is ensheathed by a complex subepithelial or mesenchymal microenvironment that is composed of diverse cell types, including stromal cells, muscle cells, various fibroblast subtypes, pericytes and endothelial cells, all of which secrete multiple factors. These factors – often referred to as niche factors – instruct ISCs to proliferate and to differentiate during homeostasis and during injury-induced regeneration of the intestinal epithelium (Kim et al., 2020; Greicius et al., 2018; McCarthy et al., 2020; Stzepourginski et al., 2017; Shoshkes-Carmel et al., 2018; Valenta et al., 2016; Degirmenci et al., 2018; Holloway et al., 2021; Jardé et al., 2020). Bowel disorders, such as inflammatory bowel disease (IBD) and colorectal cancer (CRC), are linked to the defective cellular composition of the intestinal mesenchyme and/or to the abnormal production of secreted factors from this mesenchyme (Kinchen et al., 2018; Roulis et al., 2014,^ 2020^; Katajisto et al., 2008; Shao et al., 2006). This emphasises the importance of understanding the interactions between the intestinal epithelium and the individual mesenchymal cell subtypes that nurture ISCs. However, the cellular and molecular complexity of the subepithelial microenvironment of the mammalian intestine has made it difficult to identify and functionally characterise individual cells and signalling components in this system. This has been made possible only recently through technological advances in imaging, single-cell RNA sequencing, organoid co-cultures and complex mouse genetic experiments (Holloway et al., 2021; Kim et al., 2020; McCarthy et al., 2020). The simpler nature of the *Drosophila* gut and its subepithelial microenvironment, as well as the diverse and large genetic toolkit available for *Drosophila*, have contributed to key discoveries about intestinal–microenvironment interactions in this model organism (Table 1) with implications to human health and disease (Perochon et al., 2021; Tamamouna et al., 2021; Kim et al., 2021b).

**Table 1.**
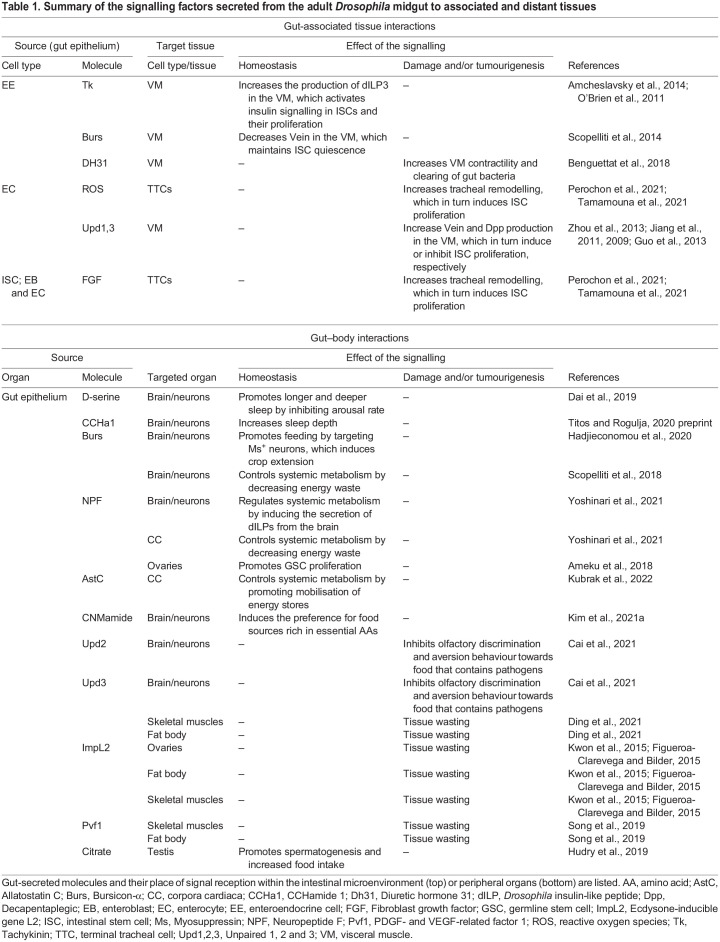
Summary of the signalling factors secreted from the adult *Drosophila* midgut to associated and distant tissues

## Intestinal–visceral muscle crosstalk in *Drosophila*

**Fig. 2.**
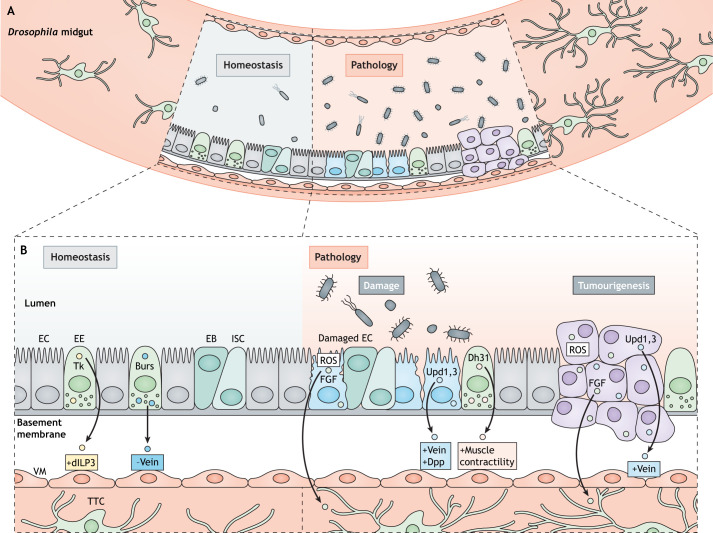
**Signalling from the adult *Drosophila* midgut to its subepithelial microenvironment*.*** (A) Schematic representation of the adult midgut epithelium and associated VM and TTCs. (B) Signalling from the intestinal epithelium to the VM and TTCs in homeostatic conditions (left), upon damage or infection (middle) and in tumorigenesis (right). In homeostatic conditions (B, left), EEs secrete Tk and Burs. Tk induces the VM to express dILP3, and Burs reduces the expression of the EGF-like ligand Vein in the VM. dILP3 promotes ISC proliferation, while repression of Vein maintains ISC quiescence. In conditions of intestinal infection or damage (B, middle), EEs secrete Dh31, which signals to the VM and induces muscle contractility to evict opportunistic bacteria from the gut. Upd3 produced by ECs stimulates the VM to secrete Vein to promote ISC proliferation. Upd1 and Upd 3 secreted by ECs also induce the release of Dpp from the VM, which restores ISC quiescence after damage. ROS and FGF from ECs activate FGF signalling in gut-associated TTCs, which induces tracheal remodelling and ISC proliferation. In tumorigenesis (B, right) Upd1 and Upd3 produced by ECs stimulate the VM to secrete Vein to promote ISC proliferation. Tumour-derived ROS and FGF activate FGF signalling in gut-associated TTCs, inducing tracheal remodelling and ISC proliferation. Burs, Bursicon-α; Dh31, Diuretic hormone 31; dILP3, *Drosophila* insulin-like peptide 3; Dpp, Decapentaplegic; EB, enteroblast; EC, enterocyte; EE, enteroendocrine cell; EGF, Epidermal growth factor; FGF, Fibroblast growth factor; ISC, intestinal stem cell; ROS, reactive oxygen species; Tk, Tachykinin; TTC, terminal tracheal cell; Upd1,3, Unpaired 1 and 3; VM, visceral muscle.

The *Drosophila* intestine is surrounded by the visceral muscle (Box 1; Fig. 2A,B), which represents the best-characterised component of the subepithelial/mesenchymal-like compartment of the adult fly midgut. In addition to its canonical role in the regulation of intestinal peristalsis, the visceral muscle has been extensively characterised for its function as the source of essential ISC niche factors, including the EGF-like ligand Vein, the Wnt ligand Wingless (Wg), JAK/STAT (also known as Hop/Stat92E) signalling ligands, BMP-like ligands and *Drosophila* insulin-like peptide 3 (dILP3; also known as Ilp3) (Biteau and Jasper, 2011; Buchon et al., 2010; Cordero et al., 2012b; Jiang et al., 2011; Lin et al., 2008, 2010; O'Brien et al., 2011; Xu et al., 2011; Guo et al., 2013). These ligands are secreted by the visceral muscle and act in a paracrine manner to activate their cognate receptors in ISCs and to induce ISC proliferation to fulfil the epithelium's demand for new differentiated cells, in response to diverse stimuli.

Egfr/Ras/MAPK signalling activity in ISCs is required to maintain the homeostatic self-renewing capacity of the intestinal epithelium (Biteau and Jasper, 2011; Xu et al., 2011). Following intestinal damage, the expression of EGF-like ligands, Vein and Spitz, are upregulated in the visceral muscle and in epithelial EBs, respectively (Jiang et al., 2011). The intramembrane protease Rhomboid, which is responsible for the cleavage and consequent activation of EGF ligands, is also upregulated in epithelial ECs, which are in close contact with EBs and the visceral muscle (Jiang et al., 2011) (Fig. 2B). This results in the activation of Egfr signalling in stem/progenitor cells and in ISC proliferation, which is required to regenerate the damaged intestinal epithelium.

The *in vivo* functional characterisation of a mesenchymal source of Wnt ligand, which is required to maintain ISC self-renewal, was first reported in the adult *Drosophila* midgut (Lin et al., 2008). Wnt from the visceral muscle appears, however, redundant for regenerative ISC proliferation, which instead depends on damage-induced epithelial Wnt/Wg, secreted by EBs in the intestinal epithelium (Cordero et al., 2012b). Follow-up work in the mammalian intestine identified similar requirements for mesenchymal or epithelial sources of Wnt ligands in intestinal homeostasis and regeneration (Suh et al., 2017; Zou et al., 2018; Aoki et al., 2016; Gregorieff et al., 2005; Valenta et al., 2016).

A key conserved pathway that has been extensively studied in the adult *Drosophila* midgut, and that is required to drive ISC proliferation and differentiation, is JAK/STAT signalling (Zhou et al., 2013; Jiang et al., 2009, 2011; Lin et al., 2010). Interleukin-like cytokines and the JAK/STAT signalling ligands Unpaired 1 and 3 (Upd1 and Upd3) are expressed in the midgut visceral muscle (Lin et al., 2010) and are highly induced in midgut epithelial ECs in response to intestinal damage or stress (Zhou et al., 2013; Jiang et al., 2009) (Fig. 2B). Although the role of muscle-derived Upd remains unclear, epithelium-derived Upd3 drives midgut regeneration by activating JAK/STAT signalling in ISCs/EBs (Jiang et al., 2009; Zhou et al., 2013). Upd3 also activates JAK/STAT signalling in the visceral muscle, which stimulates the production of the EGF-like ligands Spitz and Vein in progenitor cells and in the visceral muscle itself (Zhou et al., 2013; Jiang et al., 2011) (Fig. 2B). Spitz and Vein then activate Egfr signalling in ISCs and promote ISC proliferation. Upd1 and Upd3 released from the midgut epithelium upon damage also induce the secretion of Decapentaplegic (Dpp), a member of the bone morphogenetic protein (BMP) family, from the visceral muscle (Fig. 2B). This results in the activation of BMP signalling in the midgut epithelium (Guo et al., 2013). As in mammals, muscle-derived BMP and the subsequent activation of BMP signalling in the intestinal epithelium restrain, rather than activate, ISC proliferation (Guo et al., 2013). Therefore, JAK/STAT–BMP signalling crosstalk is key for the return of ISCs to basal proliferation levels following intestinal injury.

Intestinal carcinomas highjack microenvironmental factors and tissue-regeneration programmes to sustain their growth (Medema and Vermeulen, 2011; Ashton et al., 2010; Myant et al., 2013a,b; Cordero et al., 2014). Similarly, intestinal tumours in *Drosophila* exploit EGF-like and JAK/STAT signalling ligands, derived from the intestinal epithelium and visceral muscle, to fuel ISC hyperproliferation and tumour progression (Jiang et al., 2011; Cordero et al., 2012a; Patel et al., 2015; Song et al., 2019; Ngo et al., 2020) (Fig. 2B).

Pioneering work in *Drosophila* has revealed a pivotal role for hormone secretory EE cells in the control of intestinal homeostasis through paracrine signalling to the subepithelial microenvironment of the midgut. By releasing hormones such as Tachykinin (Tk) and Bursicon-α (Burs) (Box 1), EE cells induce insulin-like dILP3 expression (via Tk) and decrease EGF-like Vein expression (via Burs) in the visceral muscle (Amcheslavsky et al., 2014; O'Brien et al., 2011; Scopelliti et al., 2014) (Fig. 2B). dILP3 production by the visceral muscle induces insulin receptor activation in ISCs and promotes diet-induced proliferation of the midgut epithelium (Amcheslavsky et al., 2014; O'Brien et al., 2011), while the decrease in Vein in the visceral muscle, via Burs, maintains ISC quiescence in homeostatic conditions (Scopelliti et al., 2014).

Mammalian EE cells are well known for their ability to sense intestinal microbiota and microbial metabolites. This sensing capability enables EE cells to secrete hormones, which regulate visceral muscle contraction and gut motility in response to intestinal microbiota and microbial metabolites (Nozawa et al., 2009). This function of EE cells is conserved in the *Drosophila* midgut. Upon infection by opportunistic bacteria, intestinal reactive oxygen species (ROS) induce the activation of the ion channel TrpA1 in a subset of EE cells, which then secrete Diuretic hormone 31 (Dh31), an orthologue of the human calcitonin gene-related peptides (CGRPs). Dh31 binds to its receptor expressed in the visceral muscle to induce visceral muscle contraction and the clearing of gut bacteria (Benguettat et al., 2018) (Fig. 2B).

Recent findings in mice show that secretory lineage precursors, including EE precursors, are highly plastic and contribute to the homoeostatic and regenerative self-renewal of the mammalian intestinal epithelium (Tomic et al., 2018; Ishibashi et al., 2018). We anticipate that current and future findings in *Drosophila* will shed light on the mechanisms that mediate this crucial role of EE cells in the maintenance of intestinal integrity.

## Intestinal–vascular interactions in adult *Drosophila*

Endothelial cells, an integral constituent of the vertebrate vasculature, are a key component of the intestinal microenvironment (McCarthy et al., 2020; Kinchen et al., 2018; Roulis et al., 2020; Kim et al., 2020). Cellular and molecular changes in endothelial cells occur in CRC, IBD and during tissue regeneration (Perochon et al., 2021; Ippolito et al., 2016; Nolan et al., 2013; Palikuqi et al., 2020). It is, therefore, important to characterise the contribution of endothelial cells and the vasculature to adult intestinal health using *in vivo* functional studies, which currently constitutes a research area of unmet need.

Owing to the open nature of its circulatory system, the *Drosophila* model does not possess a blood-transporting vasculature, which is a key difference from mammals. However, *Drosophila* has a tracheal system akin to the mammalian respiratory and vascular systems, which consists of a branched tubular network that provides oxygen to tissues throughout the fly body (Ghabrial et al., 2003). Terminal tracheal cells (TTCs), which are equivalent to mammalian endothelial tip cells (Box 1) (del Toro et al., 2010), are highly plastic cells that can extend cytoplasmic projections towards their target tissues in order to maximise oxygen delivery. Mimicking the extensive vascularisation of the mammalian intestine, the adult *Drosophila* gut is surrounded by a dense tracheal network and represents an attractive *in vivo* paradigm for the study of intestinal–vascular interactions (Li et al., 2013; Perochon et al., 2021; Tamamouna et al., 2021).

A conserved molecular signature mediates remodelling of the developing *Drosophila* trachea and vascular remodelling and angiogenesis in mammals. Most significantly, both systems are influenced by the oxygen content of their associated tissues, which regulates the activity of *Drosophila* Similar (Sima) or of its mammalian orthologue, hypoxia-inducible factor-1α (HIF-1α) (Centanin et al., 2008, 2010; Rey and Semenza, 2010; Luo et al., 2019; Liu et al., 2018). Reduced oxygen levels activate Sima/HIF-1α, which induces the production of *Drosophila* Fibroblast growth factor (FGF; also known as Bnl) or mammalian vascular endothelial growth factor (VEGF), leading to the paracrine activation of FGF receptor (FGFR; also known as Btl) signalling in trachea (Centanin et al., 2010) or VEGF receptor (VEGFR) in the vasculature (Rey and Semenza, 2010), respectively. Developmental tracheal remodelling in *Drosophila* also depends on nutrient availability, through modulation of the insulin signalling pathway (Linneweber et al., 2014). Similarly, insulin signalling activation in endothelial cells plays important roles in mammalian vascular physiology and pathology (Vicent et al., 2003).

Recent studies have identified the cellular and molecular basis of a reciprocal crosstalk between the adult trachea and the fly midgut, which is required to induce ISC proliferation during midgut regeneration following damage (Perochon et al., 2021; Tamamouna et al., 2021) (Fig. 2B). ROS, produced by the intestinal epithelium in response to damage caused by pathogenic bacteria, activate a HIF-1α/FGF/FGFR programme in the intestinal epithelium and associated tracheal tissue, ultimately leading to intrinsic changes in gene expression within TTCs, including the production of angiocrine factors (Box 1), which are necessary to induce tracheal remodelling and ISC proliferation in the midgut (Perochon et al., 2021; Tamamouna et al., 2021). Conversely, Dpp secretion from the trachea restrains ISC proliferation in *Drosophil*a (Li et al., 2013).

Vascular remodelling is recognised as a cancer hallmark (Hanahan and Weinberg, 2000). Similarly, tumourigenesis in the *Drosophila* intestine and in other fly epithelia is associated with a substantial expansion of tracheal tissue (Grifoni et al., 2015; Tamamouna et al., 2021). Interestingly, intestinal tumours in *Drosophila* hijack regenerative midgut–tracheal signalling to induce tracheal remodelling in support of their growth (Tamamouna et al., 2021). Surprisingly, the vasculature remains a remarkably understudied component of the mammalian intestinal microenvironment. Although limitations may be imposed by the inherent differences between the *Drosophila* trachea and the mammalian circulatory system, evidence suggests that studies of midgut–tracheal interactions are likely to lead to the discovery of new biological concepts concerning the regulation and role of the vasculature, including its interaction with other components of the intestinal microenvironment, in intestinal health and disease.

Studies in mammals have demonstrated the importance of immune cells in physiological vascularisation and wound healing, and in cancer-associated angiogenesis (Fantin et al., 2010; Lin et al., 2006; Jetten et al., 2014; Lucas et al., 2010). ISC niche factors, such as Wnt ligands, are secreted by macrophages to promote mouse intestinal regeneration following injury (Saha et al., 2016). However, the role of the vasculature in macrophage-induced intestinal regeneration has not been established. In *Drosophila*, macrophage-like haemocytes promote the regenerative proliferation of ISCs in the adult midgut. They do so via the secretion of Upd3 and Dpp ligands, which activate JAK/STAT and BMP signalling, respectively (Ayyaz et al., 2015; Chakrabarti et al., 2016). Haemocytes are closely associated with the adult *Drosophila* tracheal system (Sanchez Bosch et al., 2019). Inter-organ communication studies in *Drosophila* could therefore provide a powerful *in vivo* platform in which to address immune cell–vasculature–intestinal interactions during intestinal regeneration and tumourigenesis.

## Inter-organ communication between the intestine and distant tissues

**Fig. 3.**
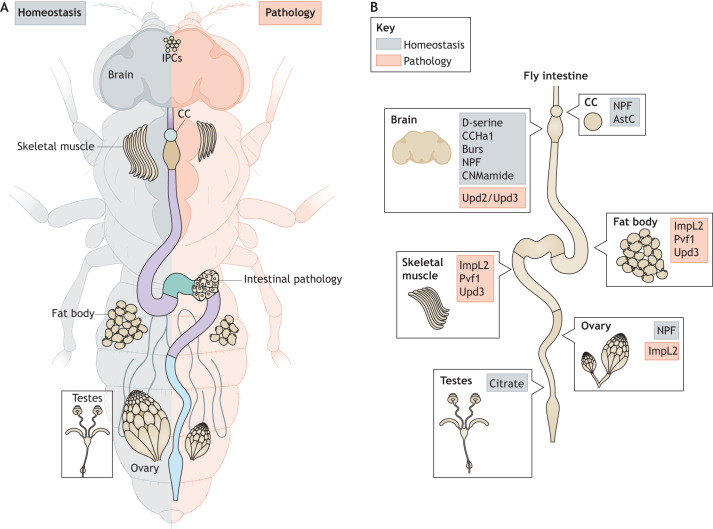
**Signalling from the adult *Drosophila* gut to distant organs in physiology and pathology.** (A) Adult *Drosophila* gut and peripheral tissues in normal physiological (left) and pathological (right) conditions. The diminished size of peripheral tissues on the right side represents organ wasting caused by signals from intestinal tumours. (B) Intestinal-derived molecules signal to peripheral tissues in physiological conditions (grey) or in pathological conditions (red). In normal physiological conditions, D-serine, CCHa1 and CNMamide, which are secreted from the gut upon nutritional inputs, regulate sleep and/or locomotor activity. EE-derived peptide hormones, Burs and NPF, signal to Myosuppressin-producing neurons and IPCs in the brain to regulate feeding behaviour and the release of dILPs, respectively. EE-derived Burs, NPF and AstC also regulate the production and release of Akh/glucagon from the CC, affecting energy storage in the fat body. Mating and nutrition affect gamete production via NPF signalling to ovaries and via citrate signalling to testes. In pathological conditions, such as enteric infection, or upon ageing, Upd2 and Upd3 are secreted from the gut and activate JAK/STAT signalling in glial cells, affecting olfactory neurons and olfaction. Upd3, Pvf1 and ImpL2 secreted from intestinal tumours induce cachexia-like wasting of skeletal muscle, fat body and ovaries. Akh, Adipokinetic hormone; AstC, Allatostatin C; Burs, Bursicon-α; CC, corpora cardiaca; CCHa1, CCHamide 1; dILPs, *Drosophila* insulin-like peptides; EE, enteroendocrine cell; IPCs, insulin-producing cells; ImpL2, Ecdysone-inducible gene L2; JAK/STAT, Janus kinase/signal transducer and activator of transcription; NPF, Neuropeptide F; Pvf1, PDGF- and VEGF-related factor 1; Upd2,3, Unpaired 2 and 3.

In addition to its role in digestion and nutrient absorption, the adult intestine fulfils major endocrine, metabolic and immune functions for the body (Brierley et al., 2021; Nauck et al., 1993; Rothhammer et al., 2018), which are largely achieved via complex signalling crosstalk between the intestine and distant organs. Owing to the well-recognised connection between the intestine and systemic dysfunction, including metabolic (Larraufie et al., 2019; Lund et al., 2011) and nervous system disorders (Gomez-Nguyen et al., 2021; Wan et al., 2021), a growing number of researchers are investigating the mechanisms that underlie these signalling networks. However, functional studies of inter-organ communication can be challenging in mammals due to their intricate physiology and genetic redundancy. Despite its evolutionary distance and less complex organ system, *Drosophila* has emerged as an invaluable model for studying inter-organ signalling and the regulation of whole-body function by the adult intestine in physiology and pathology (Fig. 3A,B and Table 1), which we discuss next.

## Gut–neuronal communication

The gut–brain axis is a bidirectional signalling network that communicates the gastrointestinal tract to the nervous system. An increasing amount of evidence indicates that a strong correlation exists between neurological disorders, such as stress, depression and autism, and intestinal dysfunction (Wan et al., 2021; Gomez-Nguyen et al., 2021; Mengoni et al., 2021; Yuan et al., 2021). This is especially true of changes in the gut microbiota, which is considered to be a major mediator of the gut–brain axis. The gut microbiota generate chemical signals that act on the central nervous system. These signals are either secreted into the bloodstream and reach the brain by crossing the blood–brain barrier, or they act indirectly, through signalling via intestinal cells, immune cells or enteric neurons (Wikoff et al., 2009; Berer et al., 2011; Buckley et al., 2020). Microbial signals exert long-range effects by targeting neuronal cell function and altering host physiology and behaviour (Morais et al., 2021). Numerous recent reviews describe in detail the role of the microbiota in the gut–brain axis (Morais et al., 2021; Rutsch et al., 2020; Margolis et al., 2021; Jacobson et al., 2021; Schroeder and Bäckhed, 2016). Here, we discuss what is known about how non-microbial gut-derived signals, which in some cases might be influenced by the microbiota, affect host systemic homeostasis.

Long-term observations have linked the intestine with host physiology, metabolism and behaviour. For example, locomotor activity and sleep are directly associated with organismal nutritional status. Nutrient-deprived animals are well known to be more active and to suppress sleep (Yu et al., 2016; Keene et al., 2010; Danguir and Nicolaidis, 1979; Hua et al., 2018). Recent studies in the fly gut are beginning to shed light on the mechanisms behind this universally recognised phenomenon. Amino acid (AA) consumption is a key factor in the regulation of sleep. The nonessential AA L-serine is converted in the intestine to D-serine by the enzyme Serine racemase, which is expressed in ECs. Evidence suggests that D-serine synthesised by the fly gut is a co-agonist of N-methyl-D-aspartate receptor 1 (Nmdar1), a subtype of glutamate receptor, which, when activated in the brain, promotes longer and deeper sleep by inhibiting arousal rate (Dai et al., 2019). An additional gut-controlled mechanism that modulates arousal threshold during sleep in *Drosophila* has been suggested in a recent preprint, which describes how sensing of dietary AAs by EE cells induces the production and secretion of the neuropeptide CCHamide 1 (CCHa1), which binds to its cognate receptor in dopaminergic neurons, increasing sleep depth (Titos and Rogulja, 2020 preprint) (Fig. 3B and Table 1).

EE cells are well-known mediators of sugar sensing by the intestine, a function that is conserved between *Drosophila* and mammals (Yoshinari et al., 2021; Buchanan et al., 2022; Scopelliti et al., 2018). Recent studies in *Drosophila* have delineated multi-organ signalling relays that link sugar sensing by EE cells with systemic physiological outputs. *Drosophila* Burs is produced by a subpopulation of EE cells in the adult midgut (Scopelliti et al., 2014), which, when stimulated by dietary sugars, secrete Burs into the circulation. Activation of Burs receptor, dLGR2 (also known as Rk), in neurons, leads to the impairment of glucagon-like secretion and favours the storage of energy resources in the fat body (Fig. 3B and Table 1) (Scopelliti et al., 2018), an organ analogous to the mammalian liver and adipose tissue (Gutierrez et al., 2007). A similar role in the regulation of systemic metabolism has been assigned to *Drosophila* Neuropeptide F (NPF), a midgut-derived EE peptide hormone orthologous to mammalian neuropeptide Y (NPY). In the case of NPF, its sugar-induced secretion from EE cells preserves organismal energy resources by restraining glucagon-like secretion and inducing dILP secretion from the brain (Yoshinari et al., 2021) (Fig. 3B and Table 1).

New insights from *Drosophila* suggest that the gut is capable of relaying host physiological changes to the nervous system, beyond an organism's nutritional status. Pioneering work in *Drosophila* has demonstrated that sex and reproductive state have a significant impact on gut physiology (Hudry et al., 2016; Reiff et al., 2015; Ahmed et al., 2020). Following mating, the number of differentiated cells, including Burs-secreting EE cells, undergo a significant expansion in the adult female midgut (Hadjieconomou et al., 2020). Increased levels of Burs activate its receptor on Myosuppressin (Ms; Box 1)-producing neurons in the brain (Fig. 3B and Table 1), inducing Ms release after mating. Ms neurons, which innervate the crop, promote muscle crop extension and increased food consumption (Hadjieconomou et al., 2020). This evidence suggests that the intestine and EE cell function are core regulators of the feeding behaviour that is necessary to sustain a metabolically demanding process such as reproduction. Recent work in mice has also delineated a neuronal circuit that influences sugar preference regulated by unidentified gut signals (Tan et al., 2020). The role of EE cells as nutrient sensors (Yoshinari et al., 2021; Scopelliti et al., 2018) and regulators of feeding behaviour in *Drosophila* (Hadjieconomou et al., 2020) points toward them being prime candidates in the regulation of refined feeding decisions, including dietary choices in mammals. Consistently, work published while this article was under review demonstrates that cholecystokinin (CCK)-producing EE cells in mice can differentiate between sugars and sweeteners and, in response, transduce these signals to the brain to modulate the preference for the consumption of caloric sugars (Buchanan et al., 2022).

Although EE cells are emerging as predominant translators of gut states to the brain, recent evidence has revealed new, unsuspected players in this essential intestinal role. Germ-free or AA-deprived flies undergo increased expression of the neuropeptide CNMamide in midgut ECs, which activates CNMamide receptor-expressing neurons, inducing the animal's preference for food sources that are rich in essential AAs (Kim et al., 2021a) (Fig. 3B and Table 1). Furthermore, conditions that lead to a chronic inflammatory state in the *Drosophila* intestine, such as ageing and infection, induce the expression and release of the JAK/STAT signalling ligands Upd2 and Upd3 from midgut ECs, leading to the activation of JAK/STAT signalling in ensheathing glial cells (EGs). EGs communicate with olfactory neurons, and activation of JAK/STAT signalling in these cells inhibits olfactory discrimination and aversion behaviour towards food that contains pathogens (Cai et al., 2021) (Fig. 3B and Table 1).

Overwhelming evidence associates intestinal function with behavioural responses and emotional states (Wan et al., 2021; Gomez-Nguyen et al., 2021; Mengoni et al., 2021; Yuan et al., 2021). Reciprocally, enteric nervous system (ENS) disorders such as Hirschsprung's disease, Parkinson's disease and autism are associated with defects in gut motility (Chaidez et al., 2014; Cersosimo et al., 2013; Bethell et al., 2016). Despite recent progress in the cellular characterisation of the mammalian ENS (Drokhlyansky et al., 2020), and the significant work being done on the involvement of the microbiota in the gut–brain axis (Jacobson et al., 2021; Margolis et al., 2021; Morais et al., 2021; Rutsch et al., 2020), the mechanistic basis of these phenomena remains largely unknown.

*Drosophila* has provided invaluable insights into the role of the ENS in intestinal stem/progenitor cell differentiation (Han et al., 2015), epithelial integrity (Kenmoku et al., 2016), and the control of fluid homeostasis, excretion (Cognigni et al., 2011; Dus et al., 2015) and food consumption (Cognigni et al., 2011; Hadjieconomou et al., 2020; Oh et al., 2021; Olds and Xu, 2014; Wang et al., 2020) by the intestine. Neurological disorders have been successfully modelled in *Drosophila* (Mizuno et al., 2010; Tauber et al., 2011). Therefore, fruit fly research might be of key importance for improving our mechanistic knowledge of gut–nervous system crosstalk and its implications in human pathophysiology.

## Gut–reproductive system interactions

Gut communication with the reproductive tract is highly dependent on signalling initiated in the gonads, which can be influenced by the mating status of a fly (White et al., 2021; Ahmed et al., 2020). However, changes in the microbiota, sex differences and mating are also responsible for significant gut alterations, which are in turn highly important for reproductive success (Mallott et al., 2020; Zhang et al., 2021; Ahmed et al., 2020; White et al., 2021).

A range of studies in *Drosophila* have provided invaluable insights into the reciprocal signalling between the adult gut and reproductive organs. The release of male-derived Sex Peptide (SP) in the seminal fluid during mating promotes signalling from the female gonads to the intestine, via a mechanism involving 20-hydroxy-ecdysone (20HE; also known as ImpE2) and Juvenile hormone (JH; also known as Jhe). These molecules signal to their cognate receptors in ISCs and alter gut physiology to maximise gamete production (Ahmed et al., 2020; White et al., 2021; Reiff et al., 2015; Ameku et al., 2018; Zipper et al., 2020). Mating-induced intestinal remodelling is responsible for gut growth via increases in ISC proliferation and EC number (White et al., 2021). Gut expansion is also accompanied by metabolic rewiring of ECs and by the upregulation of genes involved in fatty acid synthesis and AA uptake (White et al., 2021; Reiff et al., 2015). These changes are coupled with changes in feeding behaviour to increase food intake and preference for energy-rich diets (Hadjieconomou et al., 2020; Carvalho-Santos et al., 2020), which are essential for supporting fecundity and the energy demands of egg production.

Intrinsic changes in gut physiology caused by mating also influence gametogenesis. A new role for the conserved peptide hormone NPF has been proposed to promote germline stem cell (GSC) proliferation in the germarium via signalling from the midgut EE cells to the ovaries (Ameku et al., 2018) (Fig. 3B). Whereas virgin females retain NPF in EE cells, mated ones release the hormone into the circulation (the haemolymph) to activate NPF receptor (NPFR) in the ovaries and to induce GSC division via the activation of Dpp/BMP signalling (Ameku et al., 2018). Given the recently described role of gut-derived NPF in nutrient sensing and energy metabolism (Yoshinari et al., 2021), it is reasonable to speculate that NPF and EE cells in general may act to couple GSC proliferation and reproductive success to nutrient availability. Consistent with this idea, genetically induced tumourigenesis and age-dependent dysplasia of the fly midgut, which is more prevalent in mated females (Hudry et al., 2016; Ahmed et al., 2020), have a profound effect on gut architecture, cell differentiation and the expression of digestive enzymes (Patel et al., 2015; Karpac et al., 2013; Tauc et al., 2021). This may possibly alter nutrient uptake and the expression of gut hormones, consequently inducing organ wasting and affecting gamete production and life span (Kwon et al., 2015).

As well as the mating status in females, male gonads can also influence intestinal epithelial cell biology, to support their own needs. *Drosophila* testes control sex differences in intestinal EC carbohydrate metabolism. These differences occur due to a male-biased secretion of Upd from the testis, which induces the paracrine activation of JAK/STAT signalling in midgut ECs and leads to the upregulation of genes involved in carbohydrate metabolism and the production of tricarboxylic acid cycle intermediates, such as citrate, by the intestine (Fig. 3B). Intestinal citrate is in turn required to promote spermatogenesis and increased food intake (Hudry et al., 2019). Although some reports in the literature correlate intestinal disease with reproductive dysfunction, this remains a controversial and hugely understudied area of medical research (Mayberry and Weterman, 1986; Johnson et al., 2004; Pedersen et al., 2013), to which *Drosophila* studies could make vital contributions.

## Gut communication with other metabolic tissues

The intestine plays a key role in the control of whole-body metabolism (Table 1). Loss of intestinal epithelial homeostasis or disruption of the microbiota in mammals induce intestinal inflammation, alterations in lipid absorption, and the development of obesity and metabolic syndrome (Chassaing et al., 2014, 2015; DeBosch et al., 2014; Kaliannan et al., 2013; Li et al., 2016), which contribute to insulin resistance and type 2 diabetes. Evidence of improved glycaemic levels and of type 2 diabetes remission in obese patients that have undergone gastric bypass surgery (Pories et al., 1995) has provided one of the most iconic examples of the influence of the gut on systemic metabolic homeostasis. Both direct and indirect signalling from the intestine to metabolic organs have been reported in *Drosophila* larvae and adults. However, consistent with the focus of this Review, we only discuss here work involving the adult fly gut.

*Drosophila* is a highly informative model in which to study human metabolic disorders, including obesity, dietary-induced insulin resistance and type 2 diabetes (Birse et al., 2010; Na et al., 2013; Hirabayashi et al., 2013; Sanaki et al., 2020; Lourido et al., 2021; Pereira et al., 2018; Musselman et al., 2011). Not surprisingly, the fly intestine plays a considerable role as a master regulator of systemic metabolism, through tightly regulated and complex inter-organ communication networks, involving the gut, ovaries, corpora cardiaca (CC; Box 1), fat body, skeletal muscles and the brain (Fig. 3A,B) (Meschi and Delanoue, 2021; Chatterjee and Perrimon, 2021; Carvalho-Santos et al., 2020). This inter-organ crosstalk is highly dependent on organismal nutritional status and the secretion of hormones by specialised organs. The CC produces glucagon-like Adipokinetic hormone (Akh), which, together with *Drosophila* dILPs produced by neurons in the brain, plays a pivotal role in regulating glucose and lipid mobilisation and in energy storage in the fat body (Chatterjee and Perrimon, 2021; Mattila and Hietakangas, 2017).

In *Drosophila*, the intrinsic functions of the intestinal epithelium, such as robust nutrient absorption and nutrient sensing, are crucial for regulating systemic metabolic homeostasis. EE cells play a key role as sensors of intestinal luminal content and, in response to it, secrete peptide hormones into the circulation as signals to fine-tune metabolic processes (Scopelliti et al., 2018; Yoshinari et al., 2021; Song et al., 2017). As discussed in the ‘Gut–neuronal communication’ section of this Review, EE-produced Burs and NPF peptide hormones are involved both in gut–neuronal signalling and in signalling to metabolic tissues (Yoshinari et al., 2021; Scopelliti et al., 2018) (Fig. 3B and Table 1). Furthermore, a recent article reports that the somatostatin-like peptide hormone Allatostatin C (AstC), secreted by EE cells, plays a complementary role to that of Burs and NPF in the control of systemic metabolism (Kubrak et al., 2022). The starvation-induced repression of Target of rapamycin (Tor; Box 1) increases the production and secretion of AstC by EE cells, which signals to its receptor AstC-R2 in the CC (Fig. 3B and Table 1). This in turn induces AKH secretion and the mobilisation of energy stores from the fat body to prevent hypoglycaemia and to sustain organismal well-being during nutrient stress (Kubrak et al., 2022).

The intrinsic regulation of nutrient absorption by the *Drosophila* intestine can also be controlled by local signalling from EE-derived hormones. Tk, one of the most abundant EE hormones in the *Drosophila* midgut, regulates intestinal lipid metabolism by signalling through its receptor TkR99D in ECs and by suppressing the transcription factor Sterol regulatory element binding protein (SREBP), leading to decreased midgut lipogenesis (Song et al., 2014). Depletion of Tk from EE cells results in the accumulation of lipid droplets in ECs and an increase in systemic fat content (Song et al., 2014).

Only a few of the several *Drosophila* EE-secreted peptides have a direct vertebrate orthologue, which represents a potential limitation of the model system. However, and most importantly, the biological function of EE cells is largely conserved in *Drosophila*. This includes their molecular characteristics, sensory properties and ability to signal locally and systemically (Guo et al., 2021; Gribble and Reimann, 2016; Gehart et al., 2019). Altogether, the work discussed here highlights the invaluable conceptual insights obtained from *Drosophila* on the roles of the adult intestine in controlling tissue-intrinsic and systemic metabolic homeostasis.

## Systemic effects of gut dysfunction

When the homeostasis of the intestinal epithelium is disrupted, it has a direct impact on this epithelium's key roles, including its barrier function, its nutrient absorption capacity and production of digestive enzymes, its sensing of external cues and its secretion of peptide hormones (Karpac et al., 2013; Modrzynska et al., 2021; Zhou and Boutros, 2020; Chang et al., 2017). The use of *Drosophila* as a model system in which to study gut microbiota, chronic intestinal infection, inflammation and CRC is helping to address significant gaps in our understanding of the mechanisms that mediate multiple whole-body manifestations of gut dysfunction.

Enteric bacteria provide nutrients to the host and play an important role in the modulation of local and systemic metabolism (Leitão-Gonçalves et al., 2017; Consuegra et al., 2020; Chaston et al., 2014). Microbe-free flies show an increase in intestinal and systemic lipid stores. However, this phenomenon can be reversed when flies are colonised by single bacterial strains of some species but not others, through a mechanism that involves glucose oxidation and bacterial glucose utilisation (Chaston et al., 2014). Additionally, bacterial-derived molecules, such as peptidoglycan and acetate, activate *Drosophila*’s innate immunity through the induction of Tumour necrosis factor (TNF; also known as Egr)-like immune deficiency (IMD) pathway (Box 1) in the intestine (Kamareddine et al., 2018; Charroux et al., 2018; Zugasti et al., 2020). This results in the activation of NF-κB and the production of anti-microbial peptides (Liehl et al., 2006). The long- and short-term intestinal activation of IMD due to bacterial infection or to intestinal dysbiosis (Box 1) in *Drosophila* have been associated with a decreased life span, metabolic changes in the gut and systemic organ wasting (Fig. 3A) (Chakrabarti et al., 2014; Charroux et al., 2018; Zugasti et al., 2020; Paredes et al., 2011). Although the mechanisms that underlie these phenomena have not been fully identified, increasing evidence points to a key role of the IMD pathway and EE cell-derived Tk. The microbial-derived short-chain fatty acid acetate can induce the activation of the IMD pathway in EE cells and can positively modulate the expression of Tk in the midgut (Kamareddine et al., 2018; Jugder et al., 2021). As previously mentioned in this Review, the increased expression of Tk can decrease intestinal lipogenesis (Song et al., 2014). This could be directly or indirectly associated with the depletion of energy stores in the fat body, which is also observed upon IMD activation and increased Tk production by EE cells. Interestingly, the production of ROS by Dual oxidase (Duox) in *Drosophila*, a well-known mechanism of defence against intestinal microbes, is modulated by metabolic reprograming of ECs (Chakrabarti et al., 2014; Lee et al., 2018). Duox is controlled by a signalling cascade that ultimately leads to lipid catabolism. Hence, constitutive activation of intestinal Duox could be involved in the depletion of lipids from ECs during bacterial infection (Lee et al., 2018). As Tk is known to reduce lipogenesis in ECs through SREBP (Song et al., 2014), it is likely that Tk could also be involved in the control of Duox activation.

Other intestinal pathologies, such as tumours, inflammation and age-related dysplasia, are commonly associated with systemic instability (Karpac et al., 2013; Zhou and Boutros, 2020; Chassaing et al., 2015). Hyperproliferative cells in adult fly midgut tumours compete for space in the basal membrane, promoting EC delamination and apoptosis, and driving the secretion of inflammatory cytokines (Upd1, Upd2, Upd3) and the induction of stress signalling in the intestine (Zhou and Boutros, 2020; Patel et al., 2015; Cordero et al., 2012a). These events disrupt the epithelium’s barrier function, the loss of which is linked to intestinal dysbiosis, systemic infection, systemic immune activation and metabolic alterations (Zhou and Boutros, 2020; Rera et al., 2012), which ultimately have an impact on organismal life span (Zhou and Boutros, 2020; Zhou et al., 2021). Similarly to the phenomenology associated with intestinal bacterial infection (Song et al., 2014; Kamareddine et al., 2018) and tumourigenesis (Zhou and Boutros, 2020; Kwon et al., 2015), age-related intestinal dysplasia is associated with intestinal dysbiosis, deficient intestinal lipid absorption, reduction of systemic lipid stores and systemic immune activation (Karpac et al., 2013; Guo et al., 2014).

Intestinal cancer-related systemic manifestations include the peripheral organ-wasting syndrome cachexia (Fearon et al., 2011). In contrast to anorexia, cachexia can rarely be reversed by increased feeding. Cancer patients suffering from this disorder experience poor quality of life, low response to treatment and reduced survival (Baracos et al., 2018). *Drosophila* intestinal models of cachexia have contributed to our understanding of the genetics and systemic mechanisms involved in this disorder. Hyperactivation of the *Drosophila* Yap1 orthologue, Yorkie (Yki), alone or in combination with oncogenic Ras in adult ISCs, induces midgut hyperplasia and considerable wasting of peripheral tissues, including of skeletal muscle, the fat body and the ovaries (Kwon et al., 2015; Song et al., 2019). This phenomenon is caused by secreted factors, such as the insulin antagonist Ecdysone-inducible gene L2 (ImpL2) (Kwon et al., 2015; Figueroa-Clarevega and Bilder, 2015), the inflammatory cytokine Upd3 (Ding et al., 2021) and PDGF- and VEGF-related factor 1 (Pvf1) (Song et al., 2019), which are secreted from intestinal tumours and activate signalling via their cognate receptors in peripheral tissues to induce tissue wasting (Fig. 3A,B).

Intestinal damage and tumourigenesis are also associated with significant alterations to the integrity of epithelial tissue caused by aberrant cell proliferation, cell death and defective cell differentiation (Patel et al., 2015; Tauc et al., 2021; Barresi et al., 2015; Sansom et al., 2004). The extent to which these intrinsic defects in intestinal cell homeostasis contribute to systemic effects remains unclear. *Drosophila* intestinal Yki tumours show a striking reduction in the number of EE cells and, consequently, of gut hormones. However, impaired EE cell differentiation, following the overexpression of constitutively active Notch in ISCs, had no effect on preventing tissue wasting (Song et al., 2019). This suggests that EE cell loss alone is not sufficient to induce cachectic-like, systemic wasting by the intestine. By contrast, an increase in the proportion of EE cells has been described in aging intestines (Tauc et al., 2021), upon loss of commensal microbiota (Broderick et al., 2014), and following intestinal DNA damage, oxidative stress or inflammation (Lin et al., 2010; He et al., 2018; Dai et al., 2020). Alterations in EE cells and their secreted hormones have also been observed in human IBD (Worthington et al., 2018; Harrison et al., 2013; Modrzynska et al., 2021). *Drosophila* may provide an ideal system in which to address the still-elusive role of EE cells in the pathophysiology of IBD and other intestinal disorders.

## Conclusion and perspectives

How do signals to and from the intestine integrate to sustain tissue-intrinsic and whole-body homeostasis? The work discussed here highlights the magnificent contributions that research in *Drosophila* has made towards addressing such a fundamental question.

Intestinal pathology is often associated with greatly debilitating organismal imbalance – including metabolic disease and mental illness – through largely unknown mechanisms. Although their small size and lack of a robust organoid-like system for *in vitro* growth of intestinal cells impose clear limitations for biochemical studies in the fly intestine, the future is bright for *Drosophila* as an amenable and affordable high-throughput *in vivo* platform to unravel complex signalling crosstalk between multiple tissues and organs. Furthermore, recent technologies in single-cell transcriptomics with which to analyse every organ and cell type (Li et al., 2022), as well as unparalleled options of binary genetic systems with which to temporally and spatially control gene expression, are already revolutionising the field (Deng et al., 2019; Ariyapala et al., 2020; Kockel et al., 2019; Lim et al., 2021). Additionally, pathway analysis tools (Song et al., 2019) and powerful quantitative metabolic (Scopelliti et al., 2018), physiological (Cognigni et al., 2011; Hadjieconomou et al., 2020) and behavioural (Titos and Rogulja, 2020 preprint; Leitão-Gonçalves et al., 2017) approaches equip *Drosophila* research to make ground-breaking discoveries in intestinal biology, and to further our understanding of how the intestine interacts with and influences its micro- and macro-environment in health and disease.
